# Bacteriophages as a Potential 360-Degree Pathogen Control Strategy

**DOI:** 10.3390/microorganisms9020261

**Published:** 2021-01-27

**Authors:** Maria D’Accolti, Irene Soffritti, Sante Mazzacane, Elisabetta Caselli

**Affiliations:** 1 Section of Microbiology, Department of Chemical, Pharmaceutical and Agricultural Sciences, and LTTA, University of Ferrara, 44121 Ferrara, Italy; maria.daccolti@unife.it (M.D.); irene.soffritti@unife.it (I.S.); 2 CIAS Research Centre, Department of Architecture and Chemical, Pharmaceutical and Agricultural Sciences, University of Ferrara, 44121 Ferrara, Italy; sante.mazzacane@unife.it

**Keywords:** bacteriophages, environment, surface contamination, biocontrol, antibiotic resistance

## Abstract

Bacteriophages are viruses that exclusively kill bacteria and are the most ubiquitous organisms on the planet. Since their discovery, bacteriophages have been considered an important weapon to fight human and animal infections of bacterial origin due to their specific ability to attack the associated target bacteria. With the discovery of antibiotics, phage treatment was progressively abandoned in Western countries. However, due to the recent emergence of growing antimicrobial resistance (AMR) to antibiotics, interest in phage use in human therapy has once again grown. Similarly, at the environmental level, the extensive use of disinfectants based on chemicals, including biocides in agriculture, has been associated with the emergence of resistance against disinfectants themselves, besides having a high environmental impact. Due to these issues, the applications of phages with biocontrol purposes have become an interesting option in several fields, including farms, food industry, agriculture, aquaculture and wastewater plants. Notably, phage action is maintained even when the target bacteria are multidrug resistant (MDR), rendering this option extremely interesting in counteracting AMR emergence both for therapeutical and decontamination purposes. Based on this, bacteriophages have been interestingly proposed as environmental routine sanitizers in hospitals, to counteract the spread of the pathogenic MDR bacteria that persistently contaminate hard surfaces. This review summarizes the studies aimed at evaluating the potential use of phages as decontaminants, with a special focus on hospital sanitation.

## 1. Introduction

Infections caused by bacteria were a major threat for human health throughout the centuries before the discovery of antibiotics (the so-called ‘preantibiotic era’). The discovery of penicillin by Fleming followed by the wide use of new antimicrobial molecules in the subsequent decades defined instead the so-called ‘antibiotic era’, where the diseases caused by bacteria were easily managed with success. Unfortunately, the massive and sometimes inappropriate use of antibiotics has led to the appearance, evolution and spread of mechanisms of drug resistance by which bacteria can resist and survive antimicrobial attacks, contributing to the continuous growth and diffusion of antimicrobial resistance (AMR). Especially in the last twenty years, the increase in AMR has become a global concern, so that several human pathogens have become resistant to every kind of drug (the so called ‘killer bacteria’) [1]. The World Health Organization has listed groups of bacteria particularly dangerous for the human health not so much due to their pathogenicity but rather for their AMR, which renders the infections sustained by those pathogens very difficult to treat [2]. Dues to this, the WHO also pointed out that in absence of effective actions to limit and revert such a spread, in 2050 bacterial infections might become the leading cause of death for humans, similar to what occurred in the preantibiotic era [3]. The high fraction of MDR or even pan-DR (pan-drug resistant) strains among human and animal bacterial pathogens has therefore led to an urgent need to find new strategies for MDR bacteria therapeutical treatments and/or environmental decontamination, and bacteriophages seem to be a promising tool toward achieving this aim.

Bacteriophages, also known as phages, are small viruses ranging in size from 20 to 200 nm [4], which are able to specifically infect only prokaryotic bacterial cells, thus being totally safe for humans and more generally for all the eukaryotic cells. Phages represent the most abundant and diverse biological entities in biosphere [5] and they can be easily found in all environments where bacteria grow and replicate, contributing to limiting their over-spreading and maintaining the right equilibrium in ecosystems. In fact, they are commonly detected in water, soil, sewage [6,7] and have been also isolated in human and animal samples, such as feces, urine, saliva, and serum [8,9]. Bacteriophages were first discovered in 1915 by the British bacteriologist William Twort, and, independently, in 1917 by the French-Canadian microbiologist Felix d’Herelle, who realized the existence of some biological entities possessing the ability to kill bacteria. D’Herelle named them “bacteriophages” to indicate that these viruses were able to “eat” and “devour” bacteria [10]. Like all viruses, phages are simple particles consisting of a nucleic acid genome encased in a proteinaceous capsid that protect the genetic material and help its delivery to the host prokaryotic cell. The virion display a complex morphology, not classifiable as an icosahedral or helicoidal symmetry. The vast majority of bacteriophages are classified in the order of *Caudovirales*, which are tailed viruses with a genome consisting of double stranded DNA (dsDNA) and which are grouped into three distinct families: *Myoviridae, Siphoviridae,* and *Podoviridae* [11]. Isometric, helical and pleomorphic phages represent the minority in comparison with tailed phages. Isometric phages include all four types of genome, including fragmented dsRNA (family *Cystoviridae*) whereas the helical and pleomorphic phages are mostly constituted by dsDNA genomes, with the only exception represented by the family *Inoviridae*, which are filamentous viruses with ssDNA genomes [12].

**Figure 1:**
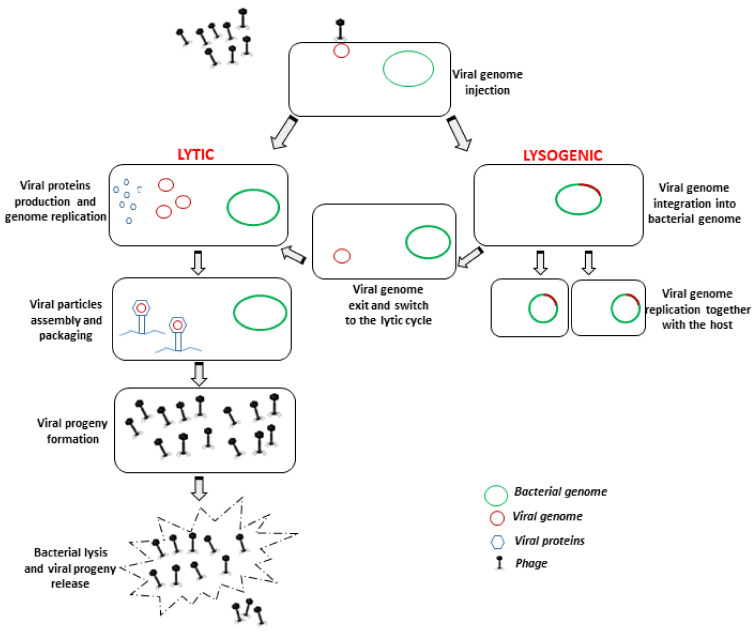
Schematic representation of bacteriophage lytic and lysogenic lifecycles, respectively carried out by virulent (lytic) and temperate (lysogenic) bacteriophages. Only lytic bacteriophages are suitable for decontamination/therapy purposes.

Bacteriophages are extremely specific towards their host, since they infect a specific species or even a specific strain, and this is determined by the nature as well as the structure of the receptors that are present on bacterial cell surfaces which interact with the phage antireceptor [13]. Once nucleic acid is injected into the host, and since they are completely free of their own molecular machinery, phages use the bacterial cell machinery to reproduce themselves, and for this reason they are defined as obligate intracellular parasites. Based on the life cycle that they can establish on the prokaryotic host, phages can be virulent or temperate (causing a lytic or lysogenic infection, respectively). The lytic cycle occurs when virulent phages infect and rapidly multiply within the host bacteria to produce viral progeny. This cycle results in the release of newly formed progeny virions by lysis of the host cell, mediated by phage-encoded enzymes able to degrade the bacterial cell wall. By contrast, temperate phages integrate their nucleic acid into the host genome, and remain in the host in a dormant stage (prophage). This type of infection is named lysogenic, and bacterial cells that are lysogenized by temperate phages acquire resistance against infection by lytic phages. The prophage can stably reside within the host cell for long periods of time until the appropriate physiological or environmental conditions favor the reactivation of the lytic lifecycle [14] (Figure 1). 

Given the antibacterial activity of lytic phages, they are considered suitable for biocontrol purposes, whereas lysogenic phages are not usable due to the high probability that they will cause horizontal gene transfer between bacteria [15], which is potentially associated with the risk of favoring the spread of AMR or other dangerous genes between microbes through bacterial transduction. However, nonlytic phages can be as very important in biomedical research, being used in phage display techniques [16], and providing the potential possibility to monitor tumors [17] and treat some diseases [18].

## 2. Phage-Based Therapy

Due to their ability to infect and lyse bacteria, lytic phages have been widely used for the treatment of bacterial infections in humans immediately after their discovery through a practice called “phage therapy”, which was especially performed in Eastern Europe. However, with the discovery of antibiotics, phage application declined after World War II, and their use continued only behind the “Iron Curtain”, in countries as Georgia or Poland [10,19].

Several old studies from USSR member states reported successful treatments of various bacterial infections, including those sustained by *P. aeruginosa* or *S. aureus*, but often these did not reach the Western World as they were written in the Russian language and did not include control groups. In fact, the paucity of peer-reviewed controlled clinical trials made it difficult to properly evaluate the effectiveness of such therapeutic by Western standards and regulations, resulting in studies that were unable to convince the Western world about phage effectiveness and safety [20].

Nevertheless, the consequences of the extensive use of antibiotics for the treatment of human as well as animal infections, leading to the uncontrolled increasing growth of AMR, contributed to the recent regrowth of interest in the use of phages, based on their interesting features. In fact, differently from wide spectrum antibiotics, phage therapy is characterized by the specificity of action, since only specific types of bacteria can be targeted by phages, with no effects on the commensal flora that keeps the host healthy [13].

Another advantage is that phage treatment does not require several administrations over repeated short times since viruses replicate on their own in the prokaryotic hosts, and for this reason very few phage doses are needed, meaning it is possible to increase their concentration at the site of infection [13,21].

Importantly, these viruses are not toxic for humans, and can eradicate biofilms thanks to the production of biofilm-degrading enzymes, rendering them effective where the antibiotic therapy fails, such as in cases of chronic infections caused by biofilms-producing bacteria [22].

On the other hand, a precise diagnosis is needed to plan a therapeutic phage application. As bacteriophages are extremely specific, wide host range phages able to infect a large number of strains are generally preferable for therapy [10]. To get this result, different phages can be mixed as cocktails to broaden the antibacterial spectrum activity, as well as to reduce the development of the resistance of bacteria to phages, since multicocktails decrease the selective pressure that can be exerted by a specific bacteriophage on its host [23]. However, in case of the appearance of bacteria resistant to phage attack, cocktails can be further modified and/or improved by adding more different phages or replacing those already present with others [22].

This procedure is quite simple, since it is possible to select phages of interest from an existing bacteriophage collection, or by isolating new bacterial viruses from the environment, these being the most abundant entities on our planet and easily findable in places with high bacterial concentrations [22,23]. Importantly, as mentioned above, only lytic phages are indicated for phage therapy, as lysogenic phages have a high probability of causing horizontal gene transfer between bacteria. Furthermore, generally only bacteriophages which have been fully sequenced are considered suitable for the treatment of bacterial diseases, since DNA sequencing guarantees the absence of lysogenic or toxic genes [15].

During the long history of using phages as therapeutic agents in Eastern Europe and the former Soviet Union, phages have been administrated to humans in different ways, (orally, by subcutaneous injections, topically) for treatment of infected ulcers and burns, and no reports of serious side effects were described associated with their use [15]. Recently, in Europe and United States some studies have been performed in vitro in experimental animals and in humans, but several questions and problems concerning the use of bacteriophages as human therapy are not solved yet, including the risk of emergence of bacteria resistant to phages, or the reduction in activity due to the immune response reaction [21]. This explains the reason why this therapy has still not been registered for clinical use in Western world. However, due to the rising emergence of AMRs, recently well-designed clinical trials were launched, mainly for wound infection in burn patients, ulcers and chronic otitis [24,25,26]. In France, Belgium and the Netherlands in 2014, the first multicenter randomized controlled trial began in patients with wound infections caused by *E. coli* showing very encouraging preliminary results [27] whereas the first clinical trial of an intravenously administered bacteriophage-based therapy has been approved in 2019 by US Food and Drug Administration (FDA) [28]. These clinical studies specifically designed to evaluate the efficacy of bacteriophages show the real intention to introduce phage therapy as part of Western clinical practices, and although they represent only the starting point, they can be useful for understanding how to prepare formulations for standardized use and studying in detail the reaction of the immune system after phage administration.

Notably, bacteriophage therapy seems to be useful in managing secondary bacterial infections in this dramatic period in which we are living.

In fact, a few weeks ago the FDA approved phage therapy for patients who present a dangerous secondary bacterial infection due to SARS-COV-2 infection, responsible for the current COVID-19 pandemic. So far, phage treatment in nine patients was shown to be helpful in reducing the multidrug resistant *Acinetobacter baumannii* infections that were present in treated patients, evidencing the potential usefulness of phage therapy in the current pandemic [29].

## 3. Food Safety-Related Applications of Phages: From Farms to Industries

Despite the skepticism toward the use of phages for human therapy in the Western World, bacteriophages have become of interest for other purposes such as alternative biocontrol strategies. Interestingly, given the high specificity of action, they have been suggested as a feasible alternative for treatment and prophylaxis in cattle, where the most prevalent infectious diseases are clinical or subclinical mastitis, metritis or respiratory infections sustained by bacterial agents [30]. For decades, antimicrobial molecules have helped to treat or prevent infectious diseases in farm animals, but their extensive utilization is associated with both environmental and human health concerns. Thus, several studies especially focused on mastitis caused by resistant strains of *S. aureus* and *E.coli* have been proposed and developed to overcome these issues, showing that phages or phage-derived products (such as lytic proteins) could effectively control these diseases [30,31,32,33].

Furthermore, phages have been proposed as decontaminating agents of food products, as many foodborne illnesses are caused by the consumption of foods contaminated by bacteria, including *E. coli* O157:H7, *Salmonella* spp. and *Listeria monocytogenes*, which represent the most dangerous foodborne pathogens [34]. Foodborne diseases today result in 420,000 deaths and an estimated 600 million cases of foodborne infections annually, representing a big worldwide concern [35]. Nevertheless, decontaminating foods (i.e., fruits, vegetables and meat) presents considerable challenges, since the strategies commonly used (washing with water or using solutions containing antibacterial chemicals) can be scarcely effective or corrosive and potentially damaging to the food itself [36]. In fact, mere treatment with water does not reduce pathogen load, and chemicals have some limits including the acquisition of resistance by microbes to the chemicals themselves and the alteration of the organoleptic properties of treated food [37]. By contrast, the biocontrol of pathogens in food products may be performed by lytic bacteriophages since they are extremely specific and act only toward bacterial cells, not representing a risk for humans and not altering food qualities [38,39].

Several studies have shown that the direct application of lytic phages to ready-to-use food can significantly reduce contamination with various foodborne pathogens [37,40,41,42,43]. Interestingly, most of them employed a cocktail of phages specific for one bacterial foodborne pathogen on food to minimize the risk of the development of resistant bacteria [44]. Based on data reported in the scientific literature, phage products have been approved by authorities for direct use on food and are commercially available.

For example, the US Food and Drug Administration (FDA), approved the use of a mixture of six bacteriophages (ListShield, by Intralytix Inc., Columbia, USA), as a direct food additive for the control of *Listeria monocytogenes* in ready-to-eat (RTE) poultry and meat products [45]. Similarly, another product called Listex P100 (manufactured by Micreos Food Safety, Wageningen, The Netherlands) and consisting of a single phage, was approved for use to inhibit the growth of *Listeria monocytogenes* in cheeses and it has received the GRAS status (generally recognized as safe). Additionally, other products, including EcoShield (Intralytix Inc., Columbia, USA), composed of three lytic bacteriophages specific for *E. coli* O157:H7, and SALMONELEX (Micreos Food Safety, Wageningen, The Netherlands) for food processing help for *Salmonella* control on beef and vegetables, are currently available.

Besides direct treatment of food, phage use is also indicated for decontaminating inanimate surfaces in farms or food-processing facilities to significantly reduce pathogen colonies on surfaces and the formation of biofilm, helping to limit the risk of transmission of pathogens along the diary chain. In fact, biofilm represents one of the most important sources of contamination with pathogens in farms or industrial settings, contributing to the transmission of pathogens to dairy products and, along the food chain, to consumers [30,46]. They are complex structures which are not easily removable by disinfectants and the resistance to antimicrobial compounds is related to several of biofilm’s intrinsic properties, including the presence of an extracellular material which constitutes a physical barrier for biocides. By contrast bacteriophages, due to their ability to produce lytic compounds and enzymes (i.e., polysaccharide depolymerases) can efficiently disrupt and prevent biofilm formation, overcoming the recognizable limits of chemical disinfectants, which are also known to be common pollutants in natural reservoirs [47], unable to prevent recontamination phenomena [48] and possibly cause potential cross-resistance with antibiotics [49].

Recent studies suggest that lytic phages can significantly reduce contamination by biofilm producing bacteria on various surfaces (e.g., gypsum board, stainless steel, glass), including *E.coli* O157:H7 [37,50], Shiga toxigenic *E.coli* O145 [51], *Listeria monocytogenes* [52], or Salmonella [53,54]. Interestingly, some products (including ListexP100 and Listshield) are consistently indicated to eliminate or reduce the levels of *L. monocytogenes* on nonfood contact equipment, thanks to their ability to prevent biofilm formation and favor biofilm elimination [55]. Consistently with more recent data, phage-based treatment in this context is becoming more and more interesting as an alternative for surface decontamination and biofilm removal in industrial settings. However, given the high specificity of these viruses and the presence of many different microbes in the microenvironment, this method seems to be less able to replace the general action of disinfectants, but rather useful for increasing their effectiveness [56]. Additionally, important concerns in phage preparation toward this use include phage propagation, purification, proper formulation and the optimization of parameters such as stability, thus limiting their general use and spread as decontaminants [56].

## 4. Bacteriophage Application in Agriculture and Aquaculture

Phages can be suitable for the control of plant pathogens that cause major economic losses in agriculture by reducing the yield and the quality of products [57]. Several studies showing phage effectiveness in crops have been published in recent years, focusing on the potential for phages to control bacterial plant diseases [58,59,60,61,62]. Based on this, a phage product (Agriphage, developed by company Omnilytics Inc., Sandy, Utah) containing specific bacteriophages against *Xanthomonas campestris* pv. Vesicatoria and *Pseudomonas syringae* bacteria responsible for bacterial spot and speck in tomatoes and peppers, respectively, has been available since 2005 [63]. In addition, a new product (AgriPhage-Fire Blight by Omnylitics Inc, Sandy, Utah and Certis USA, Columbia, USA) has been approved for us against fire blight (caused by *Erwinia amylovara*) in apples and pears since 2019 [64]. These products were shown to effectively control bacterial spot, and significantly increase yields compared to the standard copper compounds used commercially [63].

Besides agriculture, phages have also been taken into consideration for controlling infectious diseases in aquaculture, as well to control diseases associated with severe economic losses [62,65]. In fact, some bacterial species belonging to the genera *Lactococcus, Pseudomonas, Aeromonas*, and *Vibrio*, constitute the main bacterial pathogens of cultured fish and shellfish and can be also responsible for human diseases if transmitted by contaminated food [66]. Several studies have been performed in vitro and in vivo, demonstrating the effect of phages on fish bacterial pathogens, and their ability to control bacterial infections in aquaculture, even when sustained by MDR microbes [67,68,69]. Results have been obtained in the effective control of fish diseases caused by several species belonging to the genus *Vibrio* [70,71,72], or *Aeromonas* spp. [73,74], highlighting the effectiveness of phage treatment as an excellent and feasible alternative to antibiotic treatment.

## 5. Bacteriophages for Wastewater Plant Treatment

The contamination of wastewater plants by waterborne bacterial pathogens represents a global health concern, not only as a result of the consistent environmental morbidity and mortality caused, but also due to the high cost of common disinfecting methods in treatment plants, which include both physical and chemical procedures. There are a number of potential waterborne bacterial pathogens, including *Vibrio*, *Campylobacter*, *E. coli O157*, *Salmonella* and *Shigella* [75], which are known to cause several diseases with different degrees of severity, and there is an urgent need to find effective methods to counteract their growth and spread without impacting on pollution and/or AMR. In the search for ideal approaches to decrease waterborne pathogens, bacteriophages have been considered both as indicators of bacterial contamination and as good candidates for wastewater plant treatment [76]. The rationale for the use of phages as indicators is based on their specificity, thus specific bacteriophages may be used as effective tracers of pathogens in order to monitor and improve disinfection methods. The direct use of bacteriophages for decontamination purposes has instead been proposed for the elimination of filamentous bacteria in ASP (Activated Sludge Process) systems (universal aerobic treatments widely used to reduce the amount of organic matter by using microorganisms, including *Aeromonas* spp., *Pseudomonas* spp., and *Campylobacter* spp.), and for the control of foam [76,77,78,79]. However, effective application of phage biocontrol to wastewater treatment requires a complete and full understanding of the microbial communities dynamics, since the microbial population varies between different plants, and for this reason it is important to select and use specific phages able to target unwanted bacterial pathogens [77]. One obstacle to this treatment is that a high amount of phages is needed to obtain a successful result due to the complexity and size of plants’ systems, and the use of polyvalent phages with a broad range, which could have a negative effect by also targeting beneficial bacteria [76].

## 6. Bacteriophages as Environmental Sanitizers in Hospitals

Based on the decontamination of potential of phages in several contexts, their use has also been hypothesized as sanitizers in the hospital environment, as such an environment is colonized by bacteria that could potentially cause infections in the hospitalized patients. In fact, the persistent bacterial contamination of surfaces represents the major cause for the transmission of the so-called healthcare associated infections (HAIs), which are one of the most frequent and important complications for hospitalized patients in all healthcare facilities around the world. Several studies have shown that hospital surfaces are persistently contaminated by several pathogens, which can be transmitted to patients through contact and cause infections [80,81,82,83]. They include *Pseudomonas aeruginosa* [84,85], *Staphylococcus aureus* (including *Methicillin Resistant Staphylococcus Aureus*, MRSA) [86] and *Escherichia coli* [87], which are among the most frequent etiological agents of HAIs. The frequent recontamination processes, associated with the presence of colonized or infected patients, render the elimination of surface contamination a difficult task, since chemicals have some disadvantages including a temporary effect and the capability for inducing resistance against both antibiotics and chemicals [49,88]. Additionally, disinfectants kill microorganisms in an indiscriminate way, also targeting the potentially beneficial bacteria present on surfaces which usually act as “sentinels”, allowing the overspread of pathogenic and often multidrug resistant bacteria.

Interestingly, the idea of using bacteriophages as decontaminants for hard surfaces has been already explored in the literature, and some in vitro studies involving several bacterial species were developed with this aim. For example, as previously mentioned, Abuladze et al. showed that a bacteriophage cocktail containing lytic phages for *E.coli* E157:H7 was able to significantly reduce artificially contaminated hard surfaces such as glass coverslips and gypsum boards, chosen as prototypes of various hard and porous building materials respectively [37]. Similarly, cocktails of lytic bacteriophages were demonstrated to significantly reduce the number of surface-applied *Salmonella* spp. on stainless steel and glass surfaces in a study were scientists also underlined the possibility to customize phage preparations to meet the specific desired antibacterial application [54]. Moreover, in other studies phage decontamination activity was also investigated against HAI-associated pathogens, including multidrug-resistant *Acinetobacter baumannii* (MDRAB) on hard glass surfaces [89], or MRSA suggesting that phages could remove these bacteria from fomites and clothes [90].

The collected results agree about the eventual usability of bacteriophages as surface decontaminants. However, experimental conditions seem to be scarcely compatible with healthcare settings in reality, since in most cases, phages were used against high bacterial densities which favor phage–bacteria encounters [90], and were diluted in high volumes of aqueous solutions, which are useful for a prolonged phage–bacteria contact but which require that surfaces remain wet for long times and are not comparable with the presence of patients in a hospital room [37].

To be predictive for routine surface sanitation in hospitals, we performed in vitro tests by evaluating bacteriophage activity on limited bacterial target amounts on different types of surfaces, so that they were similar to those that characterize hospital surfaces according to our previous observations [48] (a density of 10^2^ CFU/24 cm^2^ were spread, corresponding to 4 × 10^4^ CFU/m^2^). Additionally, we used small liquid volumes for limiting as far as possible the time of contact in solutions between phages and bacteria [91].

Our results evidenced the ability of phages to reduce the bacterial colonies on treated surfaces by up to 90 ± 8%, even when the bacterial density was relatively low. In particular, a significant decrease was already detected at 1-h post treatment by using the lower multiplicity of infection (MOI) (10 viruses: 1 bacterial cell). Furthermore, phage efficiency increased with increasing MOI (100–1000:1), as well as increasing over time, as almost no bacteria were detected after only 6 h, and this decrease was maintained until 24 h had passed. The collected results showed phage ability in decontaminating all types of hard nonporous surfaces we tested (plastic, glass and ceramic tiles, representing the common surface types in nosocomial environments), without any significant difference between surface types and bacterial strain. Importantly, phages were able to lyse both ATCC strains and MDR hospital isolates, showing the ability of removing pathogens even when their levels were similar to those detected on nosocomial surfaces.

In addition to several in vitro studies present in the literature, the potential use of phages as decontaminating agents in the hospital environment was also investigated in the field in an interesting paper published in 2016. The study was performed in Intensive Care Units (ICUs) where bacteriophage treatment was evaluated against *Acinetobacter* spp. in addition to chemical-based conventional disinfection. In that context, a single treatment with anti- *Acinetobacter* phages reduced the occurrence of HAIs caused by such bacteria, suggesting that phages might be used effectively to reduce specific pathogens in hospital rooms. However, the aerosol phage application used was only compatible with sporadic use, when the room was empty (in other words, during terminal cleaning).

In studies evaluating the actual potential of phages to be used as decontaminants during routine hospital cleaning, their usability was investigated when additioned to ecofriendly detergents (Probiotic Cleaning Hygiene System (PCHS)) [91,92]. In particular, the tested detergent also contained nonpathogenic probiotic bacteria belonging to the *Bacillus* genus, and it was used for many years for the routine cleaning of surfaces in several Italian hospitals. The idea of a combined system originated from previous results showing the ability of probiotic cleaning to modulate the hospital microbiome, and thus the capacity of phages to ameliorate and speed up such modulation was tested. The probiotic cleaning system has already been shown to be effective [88,93,94,95,96,97] and safe for hospitalized patients [88,94,98] by inducing a gradual and stable abatement of pathogen contamination on hospital surfaces [48,88,95], including MDR pathogens [88,94], and finally inducing a significant decrease in the risk of acquiring nosocomial infections [94], in antimicrobial consumption and in associated costs [93,95]. Since PCHS action, being based on competitive exclusion mechanisms, was slow and gradual, the phages seemed good candidates to speed up the process of microbiome remodulation on treated surfaces, and were thus tested in vitro and in situ. In fact, a targeted action would be desirable in particular situations such as during outbreaks of bacterial infections, or when infected/colonized patients may increase the risk of transmission of bacterial infections to other patients, a phenomenon that is widely documented [99].

**Figure 2:**
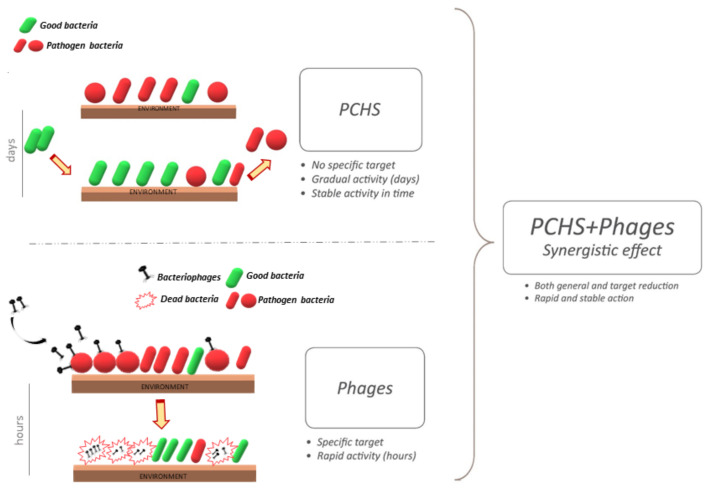
Schematic representation of combined cleaning system including a probiotic-based detergent additioned with bacteriophages. The probiotic component is characterized by a slow and gradual action, based on a mechanism based on competitive exclusion, remodulating the microbiome in a slow and gradual activity. Treatment is more rapid and specific.

On the other hand, bacteriophages are not particularly resistant in a dry environment, therefore the decontamination would be unlikely to originate a stable abatement of the targeted pathogens. Based on the complementary characteristics of bacteriophages and probiotics, a potential synergistic effect of a combined system was therefore hypothesized (Figure 2).

The results of the in vitro tests showed that phages were able to maintain their full stability when diluted in PCHS, rendering them suitable for their use as a daily sanitation system [91]. Importantly, phages could also target MDR bacteria, which are also often resistant to disinfectants [49,100]. The in situ tests consistently showed that the combined probiotic–phage application resulted in a stronger and faster decontamination activity compared to the individual probiotic and phage components alone, highlighting their possible synergistic effect [91].

To prove the potential effectiveness of phage decontamination in the hospital environment, a monocentric study was performed testing the effectiveness of the combined cleaning procedure in the ward bathrooms and targeting *Staphylococcus* spp., the bathrooms being the most contaminated areas in the hospital and Staphylococci the most prevalent bacteria in such settings, as detected by preliminary tests. The results showed that a daily combined sanitation induced a rapid and extremely significant decrease in *Staphylococcus* spp. load on treated surfaces, up to 97% more than PCHS alone [92]. A potential limitation of phage application might consist in the onset of phage resistance in treated bacteria. However, phage resistance is usually observed in phage therapy models, characterized by a high density of active proliferating bacteria [101]. Thus, with the use of phages in conditions where bacterial density is very low (such as on hospital surfaces), the onset of resistance is an unlikely event. However, further studies are needed to address this point.

## 7. Conclusions

The worldwide increase in antimicrobial resistance led scientists to search for alternative treatments to antibiotics to counteract human and animal infections. The use of pesticides in agriculture, or disinfectants for the cleaning of surfaces, are associated with environmental pollution in addition to the emergence of resistance to chemicals themselves. With the aim of overcoming these issues, lytic bacteriophages have been considered as alternative tools to counteract bacterial spread, especially of MDR strains, without impacting on environmental pollution or AMR.

**Figure 3:**
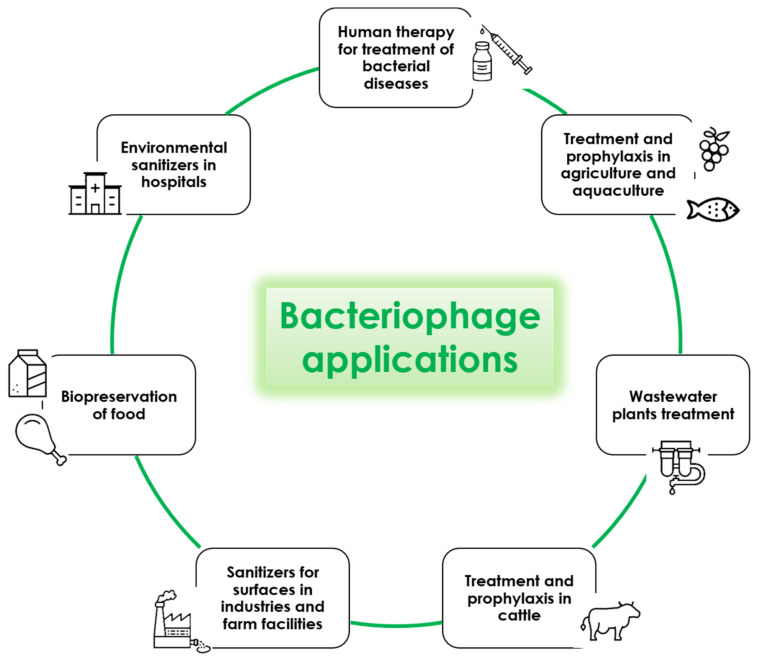
Potential applications of lytic bacteriophages.

In the scientific literature, several studies report the potential use of bacteriophages as a biological control strategy in several contexts including medicine, agriculture, food-related industries and wastewater plants (Figure 3). The results seem very promising, despite some technical issues that should be solved before phages can be extensively used as decontaminants. However, their use is already approved and in place against food pathogens, and has been tested successfully against many other bacteria responsible for animal and human infections. Lastly, interesting results were obtained by using bacteriophages for hospital cleaning, showing a very significant potential for targeted decontamination against specific pathogens. This could potentially help to prevent the persistence of targeted pathogens, consequently diminishing the risk of contracting the associated infections. Taken together, the reported results open the way to new and interesting perspectives for improving human health and simultaneously obtaining a healthier environment. This approach would also meet the “One Health” indications aimed at achieving optimal health outcomes recognizing the real interconnection between animals, people, plants and their shared environment.
